# FAITH AND HEALTH: THE INTRICATE INTERPLAY OF MARITAL QUALITY, RELIGIOSITY, AND DIABETES

**DOI:** 10.1093/geroni/igad104.2535

**Published:** 2023-12-21

**Authors:** Rebekah Case, Jeremy Yorgason

**Affiliations:** Brigham Young University, Provo, Utah, United States; Brigham Young University, Provo, Utah, United States

## Abstract

Although chronic illness in later life can negatively affect marital quality and life satisfaction through increased stress and burden, religiosity and spirituality are known robust protective factors in relational and psychological outcomes. Stress theory suggests that religious behaviors can help moderate this negative relationship by providing patients with a sense of meaning in illness, external social support, and healthy coping mechanisms. Using data from the Life and Family Legacies Study, we analyzed cross-sectional data from 1,210 married participants around age 70 in 2016. Using an OLS linear regression model, we found main effects which showed that type 2 diabetes among older adults was predictive of lower positive marital quality and lower life satisfaction. When religious behaviors were included in the model as a moderator, religious behaviors interacted significantly with diabetes such that individuals with type 2 diabetes who reported high religiosity had high positive marital quality, while those with low religiosity reported significantly lower positive marital quality. Findings suggest that when persons with type 2 diabetes participate regularly in church services, prayer, and scripture study they experience more positive marital quality. This indicates that religiosity may be a significant protective factor and coping tool for marital quality when chronic illness is present.

